# Optimization of enzyme parameters for fermentative production of biorenewable fuels and chemicals

**DOI:** 10.5936/csbj.201210005

**Published:** 2012-10-31

**Authors:** Laura R. Jarboe, Ping Liu, Kumar Babu Kautharapu, Lonnie O. Ingram

**Affiliations:** a Chemical and Biological Engineering, Iowa State University, Ames, Iowa, USA; b Microbiology, Iowa State University, Ames, Iowa, USA; c Microbiology and Cell Science, University of Florida, Gainesville, Florida, USA

**Keywords:** biofuels, biochemicals, inhibition, cofactor, affinity, transport

## Abstract

Microbial biocatalysts such as *Escherichia coli* and *Saccharomyces cerevisiae* have been extensively subjected to Metabolic Engineering for the fermentative production of biorenewable fuels and chemicals. This often entails the introduction of new enzymes, deletion of unwanted enzymes and efforts to fine-tune enzyme abundance in order to attain the desired strain performance. Enzyme performance can be quantitatively described in terms of the Michaelis-Menten type parameters K_m_, turnover number k_cat_ and K_i_, which roughly describe the affinity of an enzyme for its substrate, the speed of a reaction and the enzyme sensitivity to inhibition by regulatory molecules. Here we describe examples of where knowledge of these parameters have been used to select, evolve or engineer enzymes for the desired performance and enabled increased production of biorenewable fuels and chemicals. Examples include production of ethanol, isobutanol, 1-butanol and tyrosine and furfural tolerance. The Michaelis-Menten parameters can also be used to judge the cofactor dependence of enzymes and quantify their preference for NADH or NADPH. Similarly, enzymes can be selected, evolved or engineered for the preferred cofactor preference. Examples of exporter engineering and selection are also discussed in the context of production of malate, valine and limonene.

## Introduction

In the time since *Escherichia coli* was first engineered to produce ethanol as its major fermentation product [1] and the coining of the term “metabolic engineering” in that same year [2, 3], a variety of microbes have been engineered for the production of a wide range of products. These products include, but are not limited to, fuels [4], chemicals [5] and neutraceuticals [6]. Here we focus on the use of microbial biocatalysts to produce biorenewable fuels and chemicals.

Metabolic Engineering is defined as “the directed improvement of production, formation or cellular properties through the modification of specific biochemical reactions or the introduction of new ones with the use of recombinant DNA technology” [7]. Straightforward expression of a new pathway is often sufficient for production of the desired compound. However, an economically viable process requires that the target compound be formed at high titer (concentration), yield and rate, where the target values for these parameters can obviously vary according to the value of the product. Deletion of competing pathways and increasing expression of the target pathway are standard tools for increasing titer, yield and rate [8]. A variety of tools exist for increasing gene and enzyme abundance including the use of inducible promoters [9–12], engineering or evolution of the promoter and ribosome binding region [13], mutation of transcriptional regulators [14], transcript stabilization [15], optimization of translation initiation [16], codon optimization [17, 18] and others [8, 19, 20].

However, pathway function is determined by more than just the expression level of the constituent enzymes. The affinity of an enzyme for substrate(s) and/or cofactor(s), catalytic efficiency, cofactor requirements and allosteric regulation, as well as substrate uptake and product export, are all important drivers of flux through the desired pathway. Here we describe key examples where knowledge and manipulation of these parameters have enabled increased process performance in terms of the production of biorenewable fuels and chemicals. Note that it is often difficult to determine *a priori* which enzyme is limiting biocatalyst performance. There are several recent examples of methods for identifying problematic, or “bottleneck” enzymes [21–25]; this topic is not addressed in this review.

## Overview of Michaelis-Menten Parameters

The enzymatic conversion of substrate S to product P by enzyme E can be represented by the following simplified two-step reaction schematic (rxn 1)

$$\text{E}+\text{S}\overset{{\text{k}}_{1}}{\underset{{\text{k}}_{-1}}{⇄}}(\text{E}-\text{S})\overset{{\text{k}}_{\mathrm{cat}}}{\to }\text{E}+\text{P}$$

In this model, formation of the enzyme-substrate complex (E-S) is reversible, but formation of product P is irreversible. This schematic is represented mathematically by the Michaelis-Menten equation

$$\text{v}=\frac{{\text{v}}_{\max}{\text{c}}_{\text{s}}}{{\text{K}}_{\text{m}}+{\text{c}}_{\text{s}}}$$

where

$${\text{K}}_{\text{m}}=\frac{{\text{k}}_{-1}+{\text{k}}_{\mathrm{cat}}}{{\text{k}}_{1}}$$

CET=CE+CE-S

$${\text{v}}_{\max}={\text{k}}_{\mathrm{cat}}\text{CET}$$

In this manner, v reflects the overall velocity (rate) of a given reaction as a function of substrate concentration c_s_, concentration of active enzyme c_ET_, Michaelis constant K_m_, and turnover number k_cat_. Note that c_E_ and c_E-S_ represent the concentration of enzyme in the unbound and substrate-bound states, respectively. This formulation was first described in 1913 and has recently been translated into English and revisited with some interesting insights [26].

v_max_ and K_m_ are the two most-commonly quantified values for a particular enzyme-substrate pair, as they can be determined by measuring reaction rate v over a range of substrate concentrations. When the substrate concentration becomes saturating, the reaction velocity will approach v_max_. K_m_ is the substrate concentration at which the reaction velocity is one half of v_max_. Thus, K_m_ reflects the affinity of an enzyme for its substrate, with a lower value indicating a stronger affinity. k_cat_, also known as the turnover number, represents the speed at which a particular enzyme can convert substrate to product; higher values represent a faster-acting enzyme. The theoretical upper limit of k_cat_ is generally considered to be in the range of 10^6^ – 10^7^ s^−1^ [27]. The ratio of k_cat_/K_m_ is often referred to as the ‘specificity constant’ and used to compare the activity of a particular enzyme with multiple substrates; the theoretical upper limit of k_cat_/K_m_ is estimated as 10^8^-10^9^ M^−1^s^−1^ [27]. This ratio is also said to reflect an enzyme's catalytic efficiency, though there are concerns about the validity of this term [28]. A recent compilation and analysis of data for more than 1,800 enzymes reported that median values for k_cat_, K_m_ and k_cat_/K_m_ are 13.7 s^−1^, 130 µM and 125,000 M^−1^s^−1^, respectively [27].

## Impact of Michaelis-Menten parameters on biocatalyst performance

K_m_ values are especially important at metabolic nodes, where multiple enzymes compete for one substrate. When engineering *E. coli* for homoethanol production, Ohta *et al* [1] introduced pyruvate decarboxylase (PDC, K_m_^pyruvate^ = 0.4 mM) into an existing pyruvate node, where other enzymes (pyruvate formate lyase, K_m_^pyruvate^ = 2.0 mM; lactate dehydrogenase, K_m_^pyruvate^ = 7.2 mM) were already competing for pyruvate. However, PDC had the lowest K_m_^pyruvate^ and was able to effectively out-compete the other enzymes, enabling production of ethanol at 95% of the theoretical yield without deletion of the competing enzymes [1, 29].

Metabolic cofactors, such as ATP and NAD(P)H can be considered among the most highly-connected metabolic nodes. In these cases, enzymes with a high affinity (low K_m_) for these valuable metabolites can be problematic for a well-performing strain if these enzymes are not involved in product formation. For example, *E. coli*'s YqhD is an NADPH-dependent promiscuous aldehyde reductase that normally functions to reduce the toxic aldehydes that are produced by lipid peroxidation [30]. It has a K_m_^NADPH^ of 0.8 µM [29, 31]. However, in the presence of exogenous aldehydes, such as the furfural that can be relatively abundant in hydrolyzed biomass, YqhD-mediated furfural reduction results in depletion of the NADPH pool [31, 32]. This depletion is so extreme that there is insufficient NADPH for sulfite reductase (K_m_^NADPH^ = 80µM) to produce the hydrogen sulfide required for production of cysteine [31, 32]. This depletion of cysteine results in a lack of growth and therefore a lack of product formation. Elimination of this NADPH depletion via silencing or removal of *yqhD* results in increased furfural tolerance, both in terms of biocatalyst growth and product formation [31, 32].

A high K_m_ value can be problematic when it results in incomplete substrate utilization. A demonstration of this problem is the levoglucosan kinase (LGK) enzyme. Levoglucosan is an anhydrosugar produced during biomass pyrolysis that can be utilized with the same ATP and redox demand as glucose [33]. However, LGK has a relatively high K_m_^levoglucosan^ of 75 mM [34]. The problem incurred by this K_m_ value is reflected by the fact that a substantial amount of levoglucosan is left unutilized, resulting in a loss in product formation [33]. This problem could potentially be alleviated by modifying the enzyme to have a lower K_m_; examples of this type of modification are described below.

## Improving K_m_, k_cat_ and k_cat_/K_m_ to improve strain performance

As highlighted above, the use of enzymes with appropriate Michaelis-Menten parameters can enhance the performance of a microbial biocatalyst. The question becomes how to obtain enzymes with the appropriate parameters. In some cases, there exist characterized isozymes for a given enzymatic reaction. However, in many cases it becomes necessary to generate variants of an enzyme in order to obtain the desired function. These variants can either be generated by evolution [39–42] or through rational design [43, 44].

Chen *et al* [21] recently provided an excellent example of the how improving the Michaelis-Menten parameters of one enzyme can improve process performance. Having identified transaldolase (TAL), a component of the non-oxidative branch of the pentose phosphate pathway, as the enzyme limiting the utilization of pentose sugars by ethanol-producing *Pichia stipitis*, Chen *et al* set out to generate improved variants of this enzyme through directed evolution and screening. The most promising variant (Gln263Arg) had a two-fold decrease in K_m_^F6P^ and 3-fold increase in k_cat_^F6P^, resulting in a 5-fold increase in the k_cat_/K_m_ ratio (Table 1). When the fermentative performance of the strain expressing this improved enzyme was compared to the strain with the original TAL enzyme, an increase in both the xylose consumption rate and ethanol production rate were observed (Table 1).

**Table 1:** Improvement in fermentative performance by improving enzyme parameters.

Enzyme	Substrate	K_m_ (µM)	k_cat_ (s^−1^)	k_cat_/K_m_ (M^−1^ s^−1^)	Performance	Source
native transaldolase (TAL) in *P. stipitis* for ethanol (EtOH) production from xylose (xyl)						[21]
wild-type	F6P	560 ± 50	9.5 ± 0.7	1.7 x 10^4^	1.45 ± 0.06 g xyl consumed/L/hr 0.69 ± 0.05 g EtOH produced/L/hr	
Q263R		320 ± 10	32 ± 1	9.8 x 10^4^	1.66 ± 0.04 g xyl consumed/L/hr 0.86 ± 0.05 g EtOH produced/L/hr	
*L. lactis* alcohol dehydrogenase (AdhA) in *E. coli* 1993 + IlvC-6E6 for anaerobic isobutanol production						[35]
wild-type (his6)	isobutyr-aldehyde	11,700	30	2.8x10^3^	produced 8 g/L isobutanol	
RE1: Y50F, I212T, L264V		1,700	140	8.2x10^4^	produced 13.5 g/L isobutanol	
*P. stipitis* xylose reductase (PsXR) in *S. cerevisiae* for EtOH production from 15 g/L xyl and 5 g/L glucose						[36]
wild-type	NADH	30.5 ± 0.7	6.9 ± 0.1	2.3 x 10^5^	produced 8 g/L EtOH and 5 g/L xylitol	
	NADPH	2.5 ± 0.1	10.50 ± 0.02	4.2 x 10^6^		
R276H	NADH	17 ± 2	6.8 ± 0.3	4.0 x 10^5^	produced 11 g/L EtOH and 2.5 g/L xylitol	
	NADPH	1.7 ± 0.1	0.267 ± 0.003	1.6 x 10^5^		
ketol-acid reductoisomerase (IlvC) in *E. coli* strain 1993 for anaerobic isobutanol production						[35]
wild-type (his6)	NADH	1080	0.3	3.0 x10^2^	produced 1 g/L isobutanol	
	NADPH	40	3.6	8.7x10^4^		
6E6: A71S, R76D, S78D, Q110V	NADH	30	2.3	7.4x10^4^	produced 3 g/L isobutanol	
	NADPH	650	0.2	4.0x10^2^		
furfural reductase in ethanologenic *E. coli* LY180						
YqhD	furfural	9,000	*n/a*	*n/a*	deletion increases tolerance to furfural by sparing NADPH for biosynthesis	[31, 32]
	NADH	*n/a*	*n/a*	*n/a*		
	NADPH	8	*n/a*	*n/a*		
FucO	furfural	400 ± 200	*n/a*	*n/a*	overexpression increases tolerance to furfural by reducing furfural to furfuryl alcohol without depleting NADPH	[37, 38]
	NADH	2.7	*n/a*	*n/a*		
	NADPH	*n.d*.	*n.d*.	*n.d*		

n.d – no activity detected

n/a – not available

As part of an engineered pathway for isobutanol production, the *Lactococcus lactis* alcohol dehydrogenase (AdhA) was demonstrated as effective for converting isobutyraldehyde to isobutanol, though the K_m_ value was higher than other existing enzyme alternatives [45]. Screening of nearly 4,000 random variants identified amino acid changes that were useful in lowering the K_m_. Three of these changes were engineered into a final mutant termed RE1 [35]. RE1 showed a 10-fold decrease in K_m_, 4-fold increase in k_cat_ and thus 40-fold increase in k_cat_/K_m_ and enabled a nearly 2-fold increase in isobutanol titer (Table 1).

## Cofactor requirements

The above example of YqhD-mediated drainage of NADPH highlights the importance of this valuable cofactor. Relative to the glycolysis-associated NADH, NADPH can be relatively scarce. Therefore pathway designs in which NADPH is required for production of the target compound can suffer from a lack of NADPH availability. One method for dealing with this problem is to use transhydrogenase enzymes to intercovert NADH and NADPH [32, 35, 46–49]. Another method is to exchange NADPH-dependent enzyme activity for NADH-dependent enzyme activity, either by selecting an appropriate isozyme or by modifying the NADPH-dependent enzyme. This exchange of NADPH/NADH dependency was recently reviewed [50] and a few key examples are described here.

The reduction of furfural to the less-inhibitory furfuryl alcohol is performed by the NADPH-dependent aldehyde reductase YqhD in wild-type *E. coli* [31]. Deletion or silencing of *yqhD* increases tolerance of approximately 1.0 g/L of furfural by sparing NADPH for biosynthesis [31]. However, this results in a lack of detoxification of furfural to furfuryl alcohol. Wang *et al* [37] addressed this problem by increasing expression of the NADH-dependent furfural reductase FucO, enabling a 50% increase in furfural tolerance. Note that the K_m_^furfural^ of FucO is 0.4 mM, enabling it to outcompete YqhD's K_m_^furfural^ of 9 mM [31, 37] (Table 1), further highlighting the importance of using enzymes with appropriate K_m_ values.

Watanabe *et al* [36] used an enzyme modification approach to switch the *P. stipitis* xylose reductase (PsXR) enzyme from a preference for NADPH to a preference for NADH (Table 1). This cofactor switching was motivated by the goal to maintain redox balance with the NAD+ -dependent xylitol dehydrogenase, the enzyme which is immediately downstream of PsXR in the conversion of xylose to ethanol. The original enzyme had a 10-fold higher K_m_^NADH^ relative to K_m_^NADPH^, reflecting a 10-fold lower affinity for NADH, though the k_cat_^NADH^ was about 30% lower than k_cat_^NADPH^. By contrast, the evolved enzyme had a 10-fold lower K_m_^NADH^ relative to K_m_^NADPH^ and a 25-fold lower k_cat_^NADPH^ relative to k_cat_^NADH^. This combination of changes in K_m_ and k_cat_ means that the evolved enzyme has a 3-fold higher (k_cat_/K_m_)^NADH^ relative to (k_cat_/K_m_)^NADPH^, relative to the original enzyme's 20-fold higher (k_cat_/K_m_)^NADPH^ relative to (k_cat_/K_m_)^NADH^. Simply put, the original enzyme's preference for NADPH was evolved to a preference for NADH, where this preference is reflected in the K_m_, k_cat_ and k_cat_/K_m_ values. Use of this evolved PsXR enzyme in *S. cerevisiae* resulted in increased ethanol production from xylose and decreased formation of the side product xylitol (Table 1) [36].

Ketol-acid reductoisomerase (IlvC) is part of the engineered pathway that enables isobutanol production by *E. coli*. However, the NADPH dependence of this enzyme is undesirable. Bastian et al [35] used a structural alignment of this enzyme to identify key amino acid residues for mutagenesis with the goal of switching to NADH dependence. All beneficial mutations identified during the mutant screening were recombined in a final library and screened further. The best mutant had four mutations, a 3-fold decrease in K_m_^NADH^, 16-fold increase in K_m_^NADPH^ and 7-fold increase in k_cat_^NADH^ (Table 1). The combined effect of these mutations was 250-fold increase in k_cat_^NADH^/K_m_^NADH^, 200-fold decrease in k_cat_^NADPH^/K_m_^NADPH^ and 3-fold increase in isobutanol production (Table 1) [35]. This example demonstrates the benefit acquired when information regarding the enzyme structure and active site is available.

## Addressing enzyme inhibition (K_i_)

The Michaelis-Menten parameters described above all relate to an active enzyme, its affinity for the substrate and its speed in forming product. However, many enzymes have at least some degree of post-translational allosteric regulation which serves to fine-tune enzyme activity in response to the abundance of key metabolites. This activity control occurs in the form of both activation and inhibition; here we focus on examples of enzyme inhibition.

As with the Michaelis-Menten model of enzyme activity, there also exist quantitative models for enzyme inhibition. These describe both competitive and non-competitive inhibition. In standard cases of competitive inhibition the inhibitor (I) competes with the substrate for binding to the active site, resulting in the additional reaction (rxn 2)

E+I⇄(E-I)

to the simplified schematic described above. This reversible binding is described with the inhibition parameter K_i_, which reflects the affinity of the enzyme for the inhibitor according to

$${\text{K}}_{\text{i}}=\text{CECI}/\text{CE}-\text{I}$$

and the overall velocity v of the reaction is represented by the modified Michaelis-Menten equation

$$\frac{1}{\text{v}}=\frac{{\text{K}}_{\text{m}}}{{\text{v}}_{\max}{\text{c}}_{\text{s}}}\left(1+\frac{{\text{c}}_{\text{i}}}{{\text{K}}_{\text{i}}}\right)+\frac{1}{{\text{v}}_{\max}}$$

Note that c_i_ is the concentration of the inhibitor.

By contrast, in standard cases of non-competitive inhibition, the inhibitor binds to a site distinct from the active site and this binding induces a conformational change in the enzyme that decreases enzymatic activity. Thus, in addition to Rxn 2, it is possible for the inhibitor to bind to the E-S complex; this E-S-I complex can revert to E-I by dissociation of the substrate or possibly proceed to product formation, though at a much slower rate than the E-S complex in the absence of bound inhibitor. This non-competitive inhibition is modeled

$$\frac{1}{\text{v}}=\frac{{\text{K}}_{\text{m}}}{{\text{v}}_{\max}{\text{c}}_{\text{s}}}\left(1+\frac{{\text{c}}_{\text{i}}}{{\text{K}}_{\text{i}}}\right)+\frac{1-\frac{{\text{c}}_{\text{i}}}{{\text{K}}_{\text{i}}}}{{\text{v}}_{\max}}$$

Competitive and non-competitive inhibition can be distinguished by the use of Lineweaver-Burk plots, which are not discussed here. The relevance of these equations to the current work is the fact that enzyme sensitivity to inhibition can be quantified by the parameter K_i_, where a higher value indicates decreased sensitivity to inhibition.

This regulatory control of enzyme activity presumably serves to balance metabolic flux distribution and can be problematic when one desires to produce a single metabolic product at high concentration and yield, as this can conflict with the microbial need to balance production of biomass constituents. Thus, enzyme inhibition is a problem that often needs to be addressed in the fermentative production of biorenewable fuels and chemicals.

As with the other enzyme properties described above, the problem of enzyme inhibition can often be addressed by selecting from existing characterized isozymes. For example, Shen *et al* [51] observed relatively low metabolic flux through their engineered 1-butanol and 1-propanol pathways that was presumably due to inhibition of homoserine dehydrogenase (ThrA) by threonine, where threonine is an intermediate of the engineered pathway downstream of ThrA. Replacement of the native *E. coli* ThrA with a feedback-resistant mutant (ThrA^fbr^) resulted in a more than 3-fold increase in the final titers of 1-butanol and 1-propanol (Table 2) [51]. Similarly, the use of feedback-resistant mutants of 3-deoxy-D-arabino-heptulosonate-7-phosphate (DHAP) synthase (AroG) and chorismate mutase/ prephenate dehydrogenase (TyrA) each increased tyrosine production more than 10-fold when expressed individually (Table 2) and enabled even further increases in production when expressed simultaneously (*data not shown*) [52]. Note that AroG performs the first dedicated step of the tyrosine biosynthesis pathway and is inhibited by L-phenylalanine. TyrA performs the next-to-last step in tyrosine biosynthesis and is inhibited by tyrosine.

**Table 2:** Examples of addressing enzyme inhibition

Enzyme	Inhibitor	K_i_ (µM)	Performance	Source
homoserine dehydrogenase (ThrA) in *E. coli* CRS-BuOH 31				[51]
wild-type	threonine	*n/a*	produced 50 mg/L 1-propanol, 30 mg/L 1-butanol	
ThrA^fbr^BC		*n/a*	produced >150 mg/L 1-propanol, 100 mg/L 1-butanol	
DHAP synthase (AroG) in *E. coli* K12 ∆tyrR				[52, 56]
wild-type	L-phenylalanine	*n/a*	produced 6 ± 1 mg/L L-tyrosine	
D146N		*n/a*	produced 71 ± 9 mg/L L-tyrosine	
chorismate mutase / prephenate dehydrogenase (TyrA) in *E. coli* K12 ∆tyrR				[52, 57]
wild-type	L-tyrosine	*n/a*	produced 6 ± 1 mg/L L-tyrosine	
M53I, A354V		*n/a*	produced 86 ± 9 mg/L L-tyrosine	
citrate synthase in ethanologenic *E. coli* KO11				[53, 58]
*E. coli gltA*	NADH	2.8	produced 28 g/L ethanol	
*B. subtilis citZ*		*n/a*	produced 42 g/L ethanol	
dihydrolipoamide dehydrogenase (LPD) subunit of PDH in *E. coli* W3110				[54, 55]
wild-type	NADH	1.00	produced 7.3 mM ethanol	
G354K		10.0	produced 125.8 mM ethanol	

n/a – not available

Biomass formation by ethanologenic *E. coli* KO11 was limited in defined growth media due to NADH-mediated inhibition of citrate synthase, resulting in limitation of the biomass precursor alpha-ketoglutarate and limitation of overall growth and therefore product formation [53]. Replacement of the native *E. coli* citrate synthase with an NADH-resistant isozyme from *Bacillus subtilis* resulted in a 50% increase in growth and ethanol production in the desired growth condition [53].

An alternative approach to replacing an inhibition-sensitive enzyme with an inhibition-resistant isozyme is to modify the original enzyme so that the inhibition sensitivity is reduced or eliminated. This approach was taken by Kim *et al* [54, 55] in regards to pyruvate dehydrogenase (PDH). The PDH complex is normally subject to inhibition by NADH; presumably this serves to balance generation of NADH in glycolysis and the subsequent regeneration of NAD^+^ through fermentative pathways. The lack of PDH activity during fermentative growth, when NADH is abundant, has resulted in reliance on recombinant expression of the *Zymomonas mobilis* PET pathway for ethanol production by *E. coli* [29]. However, mutations within the dihydrolipoamide dehydrogenase (LPD) subunit of PDH reduced this feedback sensitivity approximately 10-fold, resulting in a 10-fold improvement of ethanol production without dependence on the *Z. mobilis* PET pathway (Table 2).

## Appropriate transporters for substrate uptake and product export

Finally, effective pathway flux requires the presence of appropriate uptake systems for the desired substrate and effective means of excreting or sequestering the product compound.

Transporters that are discovered when searching for importers can also be useful as exporters. The *Schizosaccharomyces pombe* malate transporter Mae1 (SpMae1p) was first demonstrated as useful for malate uptake by *S. cerevisiae* [59], but was also able to support a 10-fold increase in the malate titer achieved by a malate-producing *S. cerevisiae* [60].

Product export becomes increasingly important when the target compound is inhibitory to the microbial biocatalyst. Here we discuss two examples of the selection of appropriate exporters in order to improve the microbial production of an inhibitory compound. Despite the fact that it is naturally produced by *E. coli* and is necessary for protein translation, the branched-chain amino acid valine has long been known to be toxic to *E. coli* [61]. Thus, Park *et al*'s strain design for valine production included a means to mitigate intracellular valine accumulation via overexpression of the *ygaZH* transporter [62]. This strategy increased the valine titer by nearly 50% (Table 3). The YgaZH transporter is native to *E. coli* but was not previously recognized as a valine transporter; Park *et al* identified it as a potential valine exporter due to its homology with the *brnFE* branched-chain amino acid exporter encoded by *Corneybacterium glutamicum* [62].

**Table 3:** Transporter examples

Transporter	Transporter Substrate	K_m_ (µM)	Performance	Source
valine production by engineered derivative of *E. coli* W3110 Val				[62]
wild-type	valine	--	produced 4.34 ± 0.03 g/L valine	
+YgaZH		*n/a*	produced 7.6 ± 0.2 g/L valine	
limonene production by engineered derivative of *E. coli* DH1				[63]
wild-type	limonene	--	produced ∼35 mg/L limonene	
+YP_692684		*n/a*	produced ∼55 mg/L limonene	
malic acid production by *S. cerevisiae* CEN.PK PYC2 MDH3∆SKL				[60, 64]
wild-type	malate	--	produced ∼30 mM malate	
*+S. pombe* MAE1		1,600	produced 235 ± 25 mM malate	

n/a – not available

Many biorenewable compounds are not naturally produced by the microbial biocatalyst and thus there is an absence of effective export systems. Dunlop *et al* [63] generated a library of 43 efflux pumps from 15 different microbes and selected from the mixture based on their ability to increase *E. coli*'s tolerance of limonene, among other biofuels. Introduction of the most useful pump, YP_692684 from *Alcanivorax borkumensis*, enabled an approximately 50% increase in limonene titer when expressed in an *E. coli* strain engineered for limonene production.

These three examples highlight the use of native transporters, recombinant transporters and engineered/evolved transporters to increase production of biorenewable fuels and chemicals.

## Summary and Outlook

Here we have highlighted recent examples of how improvement of enzyme parameters, as reflected in the Michaelis-Menten-type parameters K_m_, k_cat_ and K_i_, can improve the fermentative performance of a microbial biocatalyst. Each of the examples that we have described represent improved biocatalyst performance in the context of production of biorenewable fuels and chemicals. While the Michaelis-Menten is a simplified model of enzyme kinetics [26, 65–68], these parameters provide a useful quantification of enzyme properties that can be enormously valuable to other researchers when selecting enzymes during pathway design. Databases such as BRENDA [69] are a useful repository of this type of information. However, it is critical that researchers continue to quantify and report these parameters for engineered or evolved enzymes so that others can make informed choices and use these enzymes when appropriate.

There are some enzymes that are tantalizing targets for improvement in order to increase production of biorenewable fuels and chemicals, yet these enzymes remain remarkably intractable to such improvement. The most well-known example is photosynthesis pathway enzyme Rubisco, which has a low catalytic efficient and poor substrate specificity [27, 70]. A recent cross-species analysis of the evolutionary landscape for Rubisco has provided interesting insight into why it has proven so difficult to improve its function [27]. Thus, despite the fact that we have described many successful examples of improving strain performance by improving enzyme parameters, it should be noted that enzyme improvement is not always feasible. Note that others have managed to obtain (slightly?) improved Rubisco mutants [70, 71].

While this work demonstrates the impact that improved enzyme properties can have on biocatalysts, it is apparent from the literature that additional collaboration between protein engineers and metabolic engineers could result in further advances. For example, Campbell *et al* [72] and Machielsen *et al* [73] have both demonstrated the ability to switch the cofactor dependence of alcohol dehydrogenase enzymes through rational design. This ability to target specific amino acids could possibly reduce the time needed to acquire useful enzymes relative to enzyme evolution. Additionally, thorough characterization of the resulting mutants adds to our understanding of enzyme design rules and could support further advances in protein engineering. Collaboration between metabolic engineers and protein engineers could ensure that high-impact enzymes are selected for study and that the enzyme modification yields not just a useful enzyme, but also useful information that could further advance our protein engineering capabilities.

It is interesting to note that while improvement of enzyme parameters can improve strain performance, the magnitude of these improvements often differs (Table 1). For example, the Q263R mutation in the *P. stipitis* transaldolase resulted in a 5-fold increase in its k_cat_/K_m_ for F6P, but less than a 30% increase in xylose consumption and ethanol production [21]. Similarly, multiple mutations in the *L. lactis* alcohol dehydrogenase resulted in a 30-fold increase in its k_cat_/K_m_ for isobutyraldehyde, the final isobutanol titer was increased less than 2-fold [35]. This is presumably due to the fact that metabolic flux through a given pathway consists of a series of enzymatic reactions, with each enzyme have its own set of governing parameters. Improvement of the so-called “bottleneck” enzyme wil only increase the flux to the limit allowed by the next bottleneck enzyme.

While it makes sense that the fold improvement in enzyme parameters will not result in the same fold improvement in strain performance, the impact that mitigation of enzyme inhibition can have on strain performance is particularly striking (Table 2). Work with three of the examples that we have described, DHAP synthase [52, 56], chorismate mutase [52, 57] and dihydrolipoamide dehydrogenase [54, 55], resulted in a greater than 10-fold increase in product titer. While this work considers only a limited set of enzyme manipulations, it is tempting to conclude that, generally speaking, addressing enzyme inhibition should be a higher priority than improving k_cat_, K_m_ and k_cat_/K_m_.
